# Diagnostic and prognostic value of serum miR-9-5p and miR-128-3p levels in early-stage acute ischemic stroke

**DOI:** 10.6061/clinics/2021/e2958

**Published:** 2021-10-20

**Authors:** Qi Wang, Fei Wang, Fengwei Fu, Jinlin Liu, Weilu Sun, Yongqing Chen

**Affiliations:** I Neurorehabilitation Center, Beijing Rehabilitation Hospital of Capital Medical University, Beijing 100144, China.; II Department of Encephalopathy, Binzhou Hospital of Traditional Chinese Medicine, Binzhou, Shandong 256600, China.; III The Fifth Department of Internal Medicine, Gucheng County Hospital of Hebei Province, Hengshui, Hebei 253800, China.; IV Laboratory Department, Gucheng County Hospital of Hebei Province, Hengshui, Hebei 253800, China.; V Department of Neurology, Gaoqing County People's Hospital, Zibo, Shandong 256300, China.; VI Department of Neurology, Yantai Municipal Laiyang Central Hospital, Yantai, Shandong 265200, China.

**Keywords:** Acute Ischemic Stroke, Serum, mir-9-5p, mir-128-3p, Prognosis, Early Diagnosis

## Abstract

**OBJECTIVES:**

To investigate the clinical utility of serum microRNA levels (miR-9-5p and miR-128-3p) in the diagnosis and prognosis of early-stage acute ischemic stroke (AIS).

**METHODS:**

We compared the differences in serum miR-9-5p and miR-128-3p levels between patients with AIS and healthy individuals (controls). The serum levels of miR-9-5p and miR-128-3p were quantified using quantitative real-time PCR, and the association of each miRNA with AIS was determined using receiver operator characteristic curve analysis. The predictive value of these indices in the diagnosis of early-stage AIS was evaluated in conjunction with that of computed tomography findings and neuron-specific enolase levels. The prognosis of patients with AIS was evaluated three months after their discharge from hospital using the modified Rankin scale, which classifies the prognosis as either favorable or poor. Logistic regression analysis was used to analyze the correlation between miR-9-5p and miR-128-3p levels and patient prognosis.

**RESULTS:**

The serum levels of miR-9-5p and miR-128-3p were upregulated in patients with AIS relative to those in healthy individuals. A pronounced correlation was identified between serum miR-9-5p and miR-128-3p levels and patient prognosis, with high levels of both miRNAs being associated with poor patient outcomes.

**CONCLUSION:**

Assessment of serum miR-9-5p and miR-128-3p levels is important for the early diagnosis and prognosis of AIS.

## INTRODUCTION

Acute ischemic stroke (AIS) accounts for 70% of all stroke cases and is associated with markedly high patient disability and mortality rates (1). Early diagnosis and timely intervention would significantly reduce these rates and improve the prognostic outcomes and quality of life of patients with AIS (2,3). Currently, AIS is diagnosed using computed tomography (CT). However, in the early stages of the disease, CT cannot identify any abnormalities in approximately 40-50% of the patients with AIS (4). Although interleukin-6 (5,6), neuron-specific enolase (NSE) (7), glial fibrillary acidic protein (8), and 25-hydroxyvitamin D (9) are associated with the onset of AIS, additional markers are needed owing to the low specificity and sensitivity of these markers.

MicroRNAs (miRNAs)—small endogenous RNAs comprising 18-24 noncoding bases (10)—have been found to be involved in various pathophysiological processes (*i.e.*, cell proliferation, immunity, metabolism, and tumorigenesis) via the post-transcriptional regulation of mRNAs (11). miRNAs have been detected in the cerebrospinal fluid, urine, serum, and plasma samples of patients with AIS, suggesting their diagnostic value (12). Their expression levels change significantly during the pathogenesis of AIS, a process that involves platelet aggregation, endothelial dysfunction, and neuronal injury. Previous studies have found that mir-424 and miRNA-15a are closely correlated with the occurrence of stroke (13,14). Indeed, the development of AIS has been found to correlate with changes in miRNA levels in the peripheral blood of patients (15).

The miRNA miR-9-5p is likely involved in the development of AIS, although its exact role is unclear. It has been shown to attenuate ischemic stroke by directly targeting endoplasmic reticulum metallopeptidase 1 (ERMP1)-mediated endoplasmic reticulum stress (16). However, other *in vivo* and *in vitro* studies have revealed that blocking of miR-9-5p and miR-128-3p activity could result in reduced ischemic stroke-induced neuronal cell death and infract volume (17). Given that the relationship between AIS and serum miR-9-5p and miR-128-3p levels has not yet been fully explored, the present study was carried out to investigate whether assessment of the serum levels of these two miRNAs has clinical utility in the early diagnosis and treatment of this type of stroke.

## METHODS

### Study participants

Patients with AIS and healthy individuals (controls) who visited our hospital between September 2018 and September 2020 were recruited for this study. The AIS group comprised 88 patients who had been diagnosed with acute cerebral ischemia according to strict inclusion and exclusion criteria (see below). The control group comprised 88 individuals who had visited our hospital for physical examination during the same period. This study was approved by the Ethics Committee of Yantai Municipal Laiyang Central Hospital (ethical reference number ChiCTR18000180365), and all study participants provided written informed consent.

### Patient inclusion and exclusion criteria

**Figure 1:**
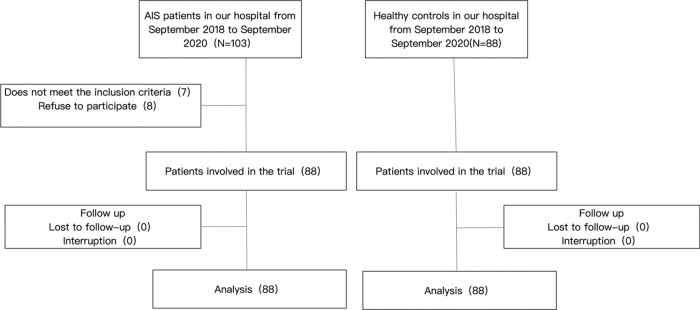
Flow chart depicting patient inclusion and exclusion.

The following inclusion criteria were employed: (i) diagnosis in strict accordance with the AIS criteria; (ii) diagnosis within 6 h after AIS onset; and (iii) confirmation of the diagnosis via CT or magnetic resonance imaging. The following exclusion criteria were employed: (i) age<18 years; (ii) comorbidities involving the heart, liver, and kidney, and other vital organ failure or insufficiency; (iii) unsigned informed consent form; and (iv) a history of cerebral hemorrhage or intracranial tumor. A flowchart of the patient-selection process has been presented in Figure 1.

### Serum preparation

Peripheral venous blood was collected from all study participants (after fasting for 12-14h). The blood samples were centrifuged at 25000×g at 4°C for 10 min, and the serum was collected and stored at -80°C.

### MiRNA isolation and quantitative real-time polymerase chain reaction (qPCR)

Total miRNA was extracted from 200 μL of each serum sample using an miRNA purification kit (Norgen Biotekand, Canada) and then reverse transcribed into cDNA using a cDNA synthesis kit (Sigma-Aldrich, USA). An miRNA qPCR kit (Sigma-Aldrich, USA) was then used to detect and quantify the miRNAs by employing a SYBR Green-based qPCR protocol. A CFX96 Real-Time PCR System (Bio-Rad, Hercules, CA, USA) was used for the qPCR. Individual miRNA-specific primers were obtained from Sino-Gene Biotechnology Ltd. (Beijing, China). The relative expression of each miRNA, which was normalized to that of the internal control *U6* small nuclear RNA, was determined using the 2^-ΔΔCT^ method (37).

### Stratified analysis

The finally selected patients were stratified into two groups based on various health indicators. Stratification according to blood pressure readings was performed based on the international diagnostic criteria for hypertension, wherein the inclusion criteria for the elevated blood pressure group were a systolic blood pressure of 140 mmHg and diastolic blood pressure of 90 mmHg. Individuals with blood pressure levels below these criteria were allocated to the normal (control) group. Stratification according to body mass index (BMI) was performed as follows: those with a BMI of 25 or higher were allocated to the high BMI group, whereas those with a BMI of less than 25 were allocated to the control group. For stratification according to low-density lipoprotein (LDL) levels, individuals with 4.1 mmol/L LDL were assigned to the elevated LDL group, whereas those with lower concentrations were assigned to the normal (control) group. The participants were also stratified into smoking and non-smoking groups, as well as groups with or without a history of hypertension and groups with or without a history of hyperlipidemia.

### Statistical analysis

All data were statistically analyzed using SPSS software (SPSS, Inc., Chicago, IL, USA) and GraphPad Prism 8. Means±standard deviations were normally distributed. Student’s *t*-test was used to perform two group comparisons. The chi-square test was used to analyze categorical variables. Receiver operator characteristic (ROC) curves were constructed, and the area under the ROC curve (AUC) was used to assess the association between each miRNA and AIS. Correlations between the serum levels of miR-9-5p and miR-128-3p and prognostic scores were analyzed using Pearson’s correlation coefficient. Differences with a *p-*value <0.05 were considered significant.

## RESULTS

### Baseline data

The patient and control groups were compared in terms of sex ratio, age, BMI, laboratory indicators (blood pressure and lipid and creatinine levels), and incidence of complications (hyperlipidemia, hypertension, and diabetes mellitus). The two groups showed significant differences in terms of blood pressure, BMI, and LDL levels (*p*<0.05). Patients in the AIS group exhibited significantly higher incidences of hyperlipidemia and hypertension than those in the control group (*p*<0.05; Table 1).

### Comparison of serum miR-9-5p and miR-128-3p levels

**Figure 2:**
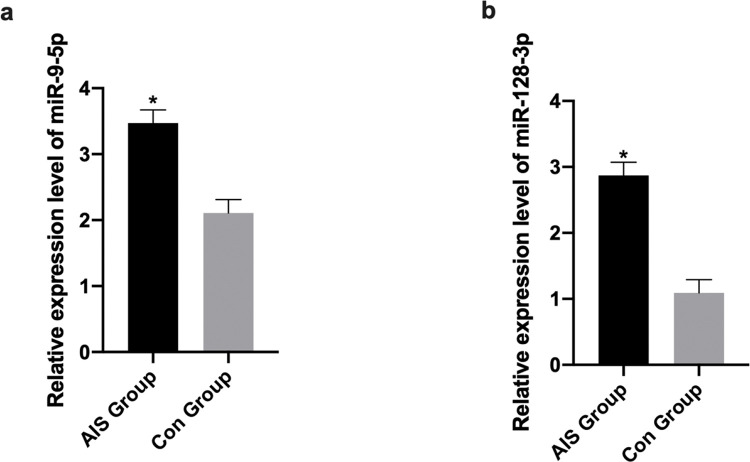
Comparison of serum miR-9-5p and miR-128-3p levels between the AIS and control groups. a) Serum miR-9-5p levels. b) Serum miR-128-3p levels. *p<0.05.

Patients in the AIS group had significantly higher serum miR-9-5p and miR-128-3p levels than those in the control group (*p*<0.05; Figure 2a, b). The levels of miR-9-5p and miR-128-3p were found to be correlated with blood pressure, BMI, LDL levels, hypertension, and hyperlipidemia. After controlling for these variables, significant differences were identified between the AIS and control groups (Tables 2 and 3).

### Detection of miR-9-5p and miR-128-3p levels via ROC curve analysis

**Figure 3:**
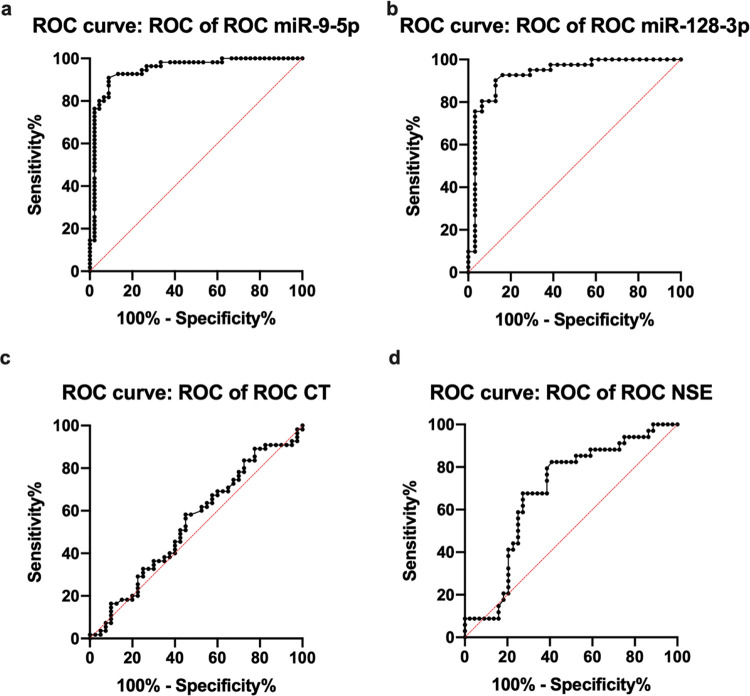
Predictive value of serum miR-9-5p and miR-128-3p levels in patients with acute ischemic stroke, as evaluated by receiver operator characteristic (ROC) curve analysis. a) ROC curve of serum miR-9-5p levels. b) ROC curve of serum miR-128-3p levels. c) ROC curve of computed tomography findings. d) ROC curve of neuron-specific enolase (NSE) levels in the serum. *p<0.05.

The relationship between serum miR-9-5p levels and AIS was confirmed by analyzing the ROC curve (Figure 3a), where the AUC was 0.9467 with a 95% confidence interval (CI) ranging from 0.8996 to 0.993. The sensitivity and specificity of the prediction were 89.75% and 82.66%, respectively. Likewise, the ROC curve (Figure 3b) confirmed the predictive value of miR-128-3p levels in the diagnosis of AIS, with an AUC of 0.9288 with a 95% CI ranging from 0.8615 to 0.9961. The sensitivity and specificity of this prediction were 73% and 79%, respectively.

The ROC curve (Figure 3c) confirmed the predictive value of CT in the diagnosis of AIS, where the AUC was 0.5423 with a 95% CI ranging from 0.4234 to 0.6612. The sensitivity and specificity were 48.45% and 55.11%, respectively. Finally, the predictive value of serum NSE levels in the diagnosis of AIS was confirmed using the ROC curve (Figure 3d), where the AUC was 0.6858 with a 95% CI ranging from 0.5650 to 0.8066. The sensitivity and specificity were 68.75% and 53.26%, respectively. Thus, miR-9-5p and miR-128-3p may serve as biomarkers for the diagnosis of AIS.

### Correlation of serum miR-9-5p and miR-128-3p levels with the modified Rankin scale (MRS) scores

The investigation of the relationship between serum miR-9-5p and miR-128-3p levels and the MRS scores revealed that the serum levels of these two miRNAs were positively correlated with the prognostic MRS scores of the patients (*p*<0.05; Table 4).

## DISCUSSION

AIS, which is the most prevalent cerebrovascular disease, has a morbidity and mortality rate of approximately 3-5% and a disability rate of 34-37% (1,18). Early diagnosis and treatment would effectively reduce the death and disability rates associated with this disease and significantly improve the quality of life of patients (19).

Brain tissues contain diverse types of miRNAs (20). Due to their very low molecular mass, miRNAs are readily secreted into the blood, and their levels in the serum are highly correlated with those in the brain tissue (21). Peripheral blood miRNAs are easily detected and have been widely used in the clinical diagnosis of various diseases because of their various advantages, such as the low invasiveness of the methods used for their analysis (22-27). In this study, the serum miR-9-5p and miR-128-3p levels were quantified using qPCR and were found to be significantly higher in patients with AIS than those in healthy individuals. Serum miR-9-5p and miR-128-3p levels exhibited a significant negative correlation with the prognostic outcomes in AIS. Therefore, the serum levels of these two miRNAs exhibit clinical utility in the early diagnosis and prevention of AIS.

Previous studies have focused on the relationships between endoplasmic reticulum stress, miRNAs, and cerebral ischemia (28). For example, miR-335 was found to be downregulated during AIS pathogenesis. Additionally, miR-335 overexpression was found to promote stress granule formation and inhibit apoptosis by targeting Rho-associated protein kinase 2 (29). The ischemic zone in rats has been shown to have reduced miR-9-5p expression. Furthermore, another study showed that the upregulation of miR-9-5p expression could improve cell viability and inhibit both lactate dehydrogenase activity and neuronal apoptosis by directly targeting ERMP1 (16). miR-9-5p expression in the brain can assist in the growth and proliferation of the nerves (30). Additionally, miR-9a-5p levels were significantly reduced in a rat model of middle cerebral artery occlusion (31). However, Sørensen et al. found that the levels of miR-9-5p and miR-128-3p were increased in the cerebrospinal fluid of patients with infarcts greater than 2 cm^3^ in volume (15). Such differences may be attributed to the different sample types used for miRNA detection. Previous studies that have compared the distribution of the same miRNA in different samples revealed that the levels are different in various types of samples and that many factors can affect their detection (32). In our study, miR-9-5p was found to be upregulated during AIS pathogenesis, which is similar to the findings of Sørensen et al. (15). We hypothesize that during the onset of acute stroke, the viability of neuronal, glial, and other cells is reduced, and in severe cases, apoptosis occurs in response to ischemia and hypoxia. By targeting B-cell lymphoma 2-like 11, miR-9-5p can inhibit neuronal apoptosis in AIS (33), and the upregulation of miR-9-5p promotes neuronal self-repair. In a comparison of 65 patients with stroke and 66 healthy individuals, researchers found a significant increase in exosome-derived miR-9 levels in the stroke group, suggesting that brain exosomes can cross the blood-brain barrier and enter the peripheral circulation (34). Other researchers found that miR-9-3p levels in the hippocampus and exosomes were increased in mice in which the central nervous system neurons had been selectively damaged, indicating the potential of this miRNA as a marker of brain injury (35). Therefore, in the early stages of stroke, detection of miR-9-5p in the blood of patients using relevant experimental technology could help clarify the diagnosis of early brain injury in stroke, and provide insights for developing efficient treatment strategies for AIS.

It has been reported that miR-128b is significantly upregulated in the plasma of patients with ischemic stroke compared to that in healthy individuals (36). Another study demonstrated a significant increase in the level of miR-128-3p in the cerebrospinal fluid of patients with AIS compared to that in the cerebrospinal fluid of the control patients (15). Our finding of a significant increase in serum miR-128-3p levels in patients with AIS is consistent with the results of the aforementioned studies. Although our results are not entirely comparable owing to the different types of samples used in various studies, our findings are supported to some extent. Therefore, in the early stages of stroke, the detection of high serum miR-128-3p levels can also be used as an important criterion for predicting the occurrence of neuronal apoptosis in patients, thus providing clues for the early diagnosis of ischemic stroke.

Our study has several limitations. First, the severity of AIS was not categorized, which may have led to study bias. Second, our cohort of patients with AIS was small; therefore, further validation in a larger multicenter study is warranted. Finally, the patients need to be followed up for a prolonged period.

In conclusion, serum miR-9-5p and miR-128-3p levels exhibit clinical utility in the early diagnosis of acute cerebral infarction and can therefore be used as potential markers for the diagnosis and treatment of AIS.

## AUTHOR CONTRIBUTIONS

Wang Q, Wang F and Chen Y conceived the project and designed and performed the experiments. Fu F, Liu J, and Sun W analyzed the data. Wang Q and Chen Y wrote and revised the manuscript.

## Figures and Tables

**Table 1:** General baseline information of the study participants.

Parameters	Control (*n*=88)	AIS (*n*=88)	*p*
Male/Female	43/45	48/40	0.661
Age (years)	62.42±3.64	63.22±5.43	0.591
Body mass index (kg/m^2^)	26.44±1.32	24.33±1.22	0.016*
Blood pressure			
Systolic pressure (mmHg)	142.33±6.13	136±2.32	0.027*
Diastolic pressure (mmHg)	94±2.77	83±2.64	0.005**
Creatinine (μmol/L)	245.09	236.22	0.521
High-density lipoprotein (mmol/L)	1.22±0.12	1.04±0.27	0.028*
Low-density lipoprotein (mmol/L)	3.28±0.28	2.61±0. 31	0.019*
Complications			
Hypertension	78	58	0.008**
Diabetes	83	47	0.003**
Hyperlipidemia	63	40	0.014*

AIS: acute ischemic stroke. *p<0.05, **p<0.01.

**Table 2:** Hierarchical analysis of miR-9-5p.

Group	Subjects (n)	miR-9-5p (mmol/L)	F	*p*
Blood pressure			9.21	<0.01**
High group	56	0.473±0.02		
Control group	32	1.023±0.03		
Body mass index			5.44	<0.01**
High group	63	0.543±0.12		
Control group	25	0.972±0.11		
Smoking			6.87	<0.01**
Yes	68	0.552±0.09		
No	20	0.927±0.05		
Low-density lipoprotein			8.44	<0.01**
High group	54	0.612±0.04		
Control group	34	0.993±0.04		
Hypertension			6.33	<0.01**
Yes	64	0.412±0.11		
No	24	0.883±0.12		
Hyperlipidemia			8.56	<0.01**
Yes	50	0.271±0.14		
No	38	1.013±0.11		

** *p*<0.01.

**Table 3:** Hierarchical analysis of miR-128-3p.

Group	Case (n)	miR-128-3p (mmol/L)	F	*p*
Blood pressure			8.33	<0.01**
High group	56	1.442±0.17		
Control group	32	1.011±0.11		
Body mass index			6.71	<0.01**
High group	63	1.553±0.10		
Control group	25	1.001±0.11		
Smoking			4.66	<0.01**
Yes	68	1.422±0.11		
No	20	1.032±0.15		
Low-density lipoprotein			7.69	<0.01**
High group	54	1.529±0.11		
Control group	34	1.021±0.13		
Hypertension			6.34	<0.01**
Yes	64	1.38±0.09		
No	24	1.04±0.12		
Hyperlipidemia			6.47	<0.01**
Yes	50	1.73±0.07		
No	38	1.022±0.12		

** *p*<0.01.

**Table 4:** Correlation of the serum miR-9-5p and miR-128-3p levels with the modified Rankin scale scores.

Parameter	*R*	*p*
miR-9-5p	0.066	0.003**
miR-128-3p	0.082	0.007**

** *p*<0.01.
